# Case Department

**Published:** 1888-10

**Authors:** 


### CASE DEPARTMENT.

### I. Notes on the Autopsy of a Chimpanzee.

### I. HISTORY.

BY W. A. CONKLIN.

*Director New York Zoological Gardens.*

The subject of this notice was known as "Mr. Crowley," and was received at the New York Zoological Gardens on the 25th of June, 1884.

His age, at the time of his reception, was judged to be about one year, and his weight fifteen pounds.

At various intervals during his life in the gardens his weight was taken and registered as follows:

| | | |
|---|---|---|
| September 11th, 1884 | 20 | pounds |
| January 15th, 1885 | 25 | " |
| May 5th, 1885 | 28 | " |
| July 16th, 1885 | $33\frac{1}{4}$ | " |
| August 24th, 1885 | $36\frac{1}{2}$ | " |
| October 20th, 1885 | $38\frac{3}{4}$ | " |
| September 25th, 1886 | 57 | " |
| June 1st, 1888 | 110 | " |

In May, 1885, he was attacked with pneumonia, from which he fully recovered. In January, 1886, he was prostrated by the same disease in a more violent form, and his recovery was apparently complete.

About the beginning of August, 1888, he suffered from constipation for some days, but was relieved by appropriate treatment. He then commenced to droop, and refused solid food. His diet was reduced to fruit and milk. He continued to fail until his death which took place on the 31st of same month.

On examination, after death, the body was found very much emaciated, the weight being only seventy-three pounds.

The following measurements were taken:

| | | |
|---|---|---|
| Total length | 4 feet, | 4 inches. |
| Trunk | 2 " | 8 " |
| Face | | 9 " |
| Ear | | 3 " |
| Arm from shoulder to end of first row of phalanges | 2 " | 8 " |
| Hand to tips of fingers | | 10 " |
| Middle finger | | $5\frac{1}{2}$ " |
| Circumference of chest | 2 " | 5 " |
| Leg | 1 " | 10 " |
| Foot | | $9\frac{3}{4}$ " |
| Foot width | | 4 " |
| Middle toe | | 3 " |

Some interesting notes on the Psycology of this animal, written by Dr. C. Pitfield Mitchell will be found published in the Journal of Comparative Medicine and Surgery, Vol. V., No. 1.

### II. SOME OBSERVATIONS OF THE BRAIN.

BY DR. EDWARD C. SPITZKA, M. D.

Dura mater; normal in texture, non-adherent, except at sutures, of a more opaque white color over the sylvian region than elsewhere.

Sinuses; filled with bluish-dark soft coagula.

Aracnoid: the lamellae transparent and healthy, marked œdema over the right sylvian, rolandic and parietal regions.

Pia; normal in texture, readily removed from cortex, and the seat of a moderate venous injection. On the upper temporal gyrus, near the apex of the temporal lobe, a lengthy area on either side of the vascular line over the sulcus was distinctly pigmented. The pigmentation was arborescent and gradually faded from the artery outwards. Similar streaks were found along the branches of the sylvian artery.

The arteries were normal, except that there were two minute white spots on the piece corresponding to the middle of the pons.

The existence of *striæ medullares albae* were determined .for the first time in the annals of anthropoid cerebral anatomy). Weight of brain, exactly 440 grammes.

### III. NOTES ON AUTOPSY.

BY WILLIAM S. GOTTHEIL, M D.

A. EXETRNAL.—1. *Inspection.—Body* much emaciated, *skin* atrophied; slight post-mortem rigidity.

B. INTERNAL.—1 *Thorax.*—a. *Panniculus adiposus* atrophied. b. *Lungs and Pleural Cavity. R. pleural cavity* obliterated; *pleura* enormously thickened, 1-6—1-2 inch thick; entire lung presents the ordinary lesions of chronic phthisis. Fibroid bands run in various directions; pea to wallnut-sized cheesy deposits are scattered all through the lung. Some are entirely softened down. At apex, a large abcess. Surrounding the deposits are zones of acutely congested lung tissue. L. *pleural cavity*; a moderate number of recent adhesions; *pleura* thickened in places. *Lung* acutely congested, studded with areas of acute inflammation. *True miliary tubercles* in moderate number in lung, and abundant on both parietal and visceral pleuræ. c. *Heart and Pericardium.* Entire visceral pericardium studded with *ecchymoses.* They are scattered over the entire surface, but are more abundantly grouped in two places, the left ventricle, at its apex, and the R. appendix. The entire muscular structure of the heart is studded with these hemorrhages, which show also upon the endocardium. They are of recent date. No free blood in the peri- or endo-cardial cavities. d. *Thoracic glands* enlarged to nut size—some dark and congested, others cheesy—and others filled with pus-like matter.

2. ABDOMEN.—Adhesions of old peritonitic process unites omentum and one or two coils of small intestine to the anterior abdominal wall; thickening of the anterior parietal peritoneum in places. a. *Liver.*—Fatty. True tubercles in quantity upon peritoneum covering organ. b. *Spleen.*—Congested. c. *Kidneys.*—Congested intensely and fatty. Number of minute hemorrhages through their substance. d. *Pelvic viscera.*—Normal. e. *Stomach and Intestinal Canal*—Studded throughout its entire length with hemorrhages. No ulcerations or free blood. f. *Mesenteric Glands.*—Much enlarged—some being small walnut-sized; capsules thickened, stroma dark and soft, or cheesy.

POST-MORTEM DIAGNOSIS.—Chronic tubercular phthisis. Cardiac and enteric hemorrhages. General tubercular adenitis.

CAUSE OF DEATH.—Exhaustion.

Rapid Union of Fracture of First Phalanx.—Called to see a bay mare that had been injured by a runaway team. Found the mare bearing no weight on left fore-leg, and the foot hanging loosely from the inferior extremity of the cannon bone, with no evidence of any abrasion of the skin whatever. On closer examination, with the presence of extreme pain, and distinct crepitus in the region of the first phalanx, the diagnosis of simple longitudinal fracture of the above said bone was made. A plaster Paris bandage was applied with splints of hickory wood and the mare placed in a sling, which she took to very kindly, though she was a mare of very nervous temperament. She was cared for by a daughter of the owner, and the benefit of constant attendance and good nursing was plainly visible. After two weeks she began to paw with the injured limb, and show symtoms of uneasiness, from some unexplainable cause.

Fearing an abcess, or the casting off of small fragments of bone, as the mare had borne considerable weight upon the limb, it was thought advisable to remove the dressing entirely, which was done on the 24th day after the fracture occurred.

Complete union of bone was found with no interference with the Metatarso phalangeal or inter-phalangeal articulations, and the cause of uneasiness due to extreme itchiness of the skin from confinement beneath the bandage. The mare would exhibit the greatest satisfaction when the part was handled, so bathing with cold water and the application of chloral hydrate removed the burning sensation of the part.

The animal continued to improve and bear more weight upon her leg, and on the thirty-fourth day after the fracture, the slings were removed. Several days later, she walked from the stable to a paddock near by, and was only slightly lame with some little swelling in the part. Fifty-six days after the accident, the mare stood for her shoes to be removed and is at present grazing leisurely and on a good road to complete recovery.

Dr. Leon N. Reefer.
Meadville, Pa. Dr. Chas. C. McLean.

---

Congenital Failure of Union of Folded and Floating Colon.—Called to see sorrel filly, which had been born day before, 9 A. M. Shortly after birth was strong and lively, nursing and playing, and apparently in best of health, well nourished and straight on limbs, well made and good size. The evening of the day of birth, attendant found it playing and nursing mare, as well as any colt possibly could. The next morning foal was found with eyes closed and skinned, as we often see in a colic of long standing, and well-marked colic symptoms, rolling and lying on back. When seen there was some tympanitis and great tenesmus. Rectal examination with finger revealed nothing, not even stain. Examination of paddock and apartment showed no foal faeces. The colt, when standing, showed clear symptoms of pressing back and stretching, as if to urinate.

Diagnosis.—Impaction of Faecal Matter in Colon.—Treatment consisted of light oleaginous purge and enema, followed by enema of glycerine (with which, by the way, I have had excellent results in exciting peristaltic motion), but, in this case, of no avail; all enema retained but colic more marked after introduction of same. I then feared a rupture of intestine, but could trace no cause, unless tympany had been excessive during night; but constitutional symptoms were not of such character as to justify such an opinion, consequently dismissed it; foal continued to fail, but the pain was no more severe, as chloral was given. At evening, pulse 175.; respirations not hurried, which is peculiar; ears, mouth and extremeties warm; colt moaning and back position desired constantly.

At 10 P. M. it had stopped rolling, but distress was marked. I had punctured colon four times, to relieve tympany and suffering. The puncture was done with hypodermic needle. At one puncture, free passage of enema water from needle, known to be so from fact of Oleum Lini, given in enema, could readily be seen in water from needle. This, of course, aroused and substantiated diagnosis of rupture as water escaped before needle penetrated bowel. A fatal prognosis had been given long before and case was abandoned. I would have destroyed colt at once if owner's consent could have been obtained. Foal died next morning.

AUTOPSY.*—On incision in median line, a large quantity of water escaped, well marked perionitis. Beginning at rectum and following intestine, came to an end at the ante ior section of floating colon, as it terminated in a *cul de sac* like the apex of cœcum. The posterior portion of colon ended in a like manner, resembling the cœcum of an ox. There was no connection of any character between the two sacs, not even a continuance of mesentary. There was absolutely no relation.

DR. CHAS. C. McLEAN.
MEADVILLE, PA. DR. LEON N. REEFER.

COMPOUND FRACTURE OF RADIUS IN TWO-YEAR-OLD FILLY—AMPUTATION AND RECOVERY.—August 24th I was called to Evansburg, Pa., to see a two-year-old filly, called "Bonnie Janet." On arrival I found the mare in a large pasture, over half a mile from the stable, with a compound fractured forearm, both ends of fracture protruding. After advising owners that the only possible means of saving the filly for a a brood mare would be amputation of the limb, they made preparaions to have her removed to stable, which was done at once. On arrival, she was cast, and sulph. eth. used for anæthesia, a rapid circular incision with long bladed knife on each side, made a good flap, and left a nice cushion, the anterior and posterior radical arteries were ligated. Radius sawed as high as flaps would allow, a large twitch was used for a tourniquet. A cold solution of mercuric bichloride subdued a slight hemorrhage of small vessels, wound thoroughly dusted with iodoform and flaps closed with strong gut and wire sutures, leaving a three inch space in middle for drainage. A cold water bandage was applied after dusting entire stump with iodoform and hobbles and rope were removed. She lay perfectly quiet for an hour and thirty minutes, except some trembling of shoulder muscles, and then made an effort to regain standing position; on the third attempt she was assisted by three very able stable men, and was at once led to a large box which had been well cleaned and disinfected. She stood for one hour and ate a hearty meal, and then laid down and rested very quietly until 12 P. M. Being of the same opinion of Prof. Huidekoper of Pennsylvania University about his amputation at Avondale:

*The intestines have been very nicely prepared and are now in the Veterinary Museum of the University of Pennsylvania.

"that the subject should be taught to depend on her herself at once." The slings were not used, except on three occasions, when she stood too long, at one time fourteen hours. They were then used to lay her down. On second day she got up without any assistance, and has continued to do so each day, and as frequently as she desired ; moves about the stall in a manner quite surprising to the many curious visitors who have called to see a three legged horse. The sutures remained twelve days, union of flaps progressed very rapidly. suppuration slight, the swelling from fracture decreased rapidly after operation, dressing consisted of cold water bandages, iodoform freely, and injections ever two hours in drainage hole of hydra per chl., 1 to 1,000 of water. Her diet consisted of green corn, apples and boiled grain liberally, and the stable surroundings were kept unusually clean. She exhibited a great degree of intelligence, on several occasions in lying down she would forget and drop to the left side, but when she got ready to rise, she will roll over on side of remaining member and rise without difficulty. Her temperature never rose above 101° F., pulse, after operation, 82 ; but day following had dropped to 64 and continued to fall to 42, at which it has remained. The mastoido-humeralis muscle has developed greatly, also all muscles of right extremity.

In conclusion I would say that I am of the opinion that many a valuable stock animal can be saved by amputation, when the fracture will not justify treatment by usual method, and that the wholesale slaughter of animals with broken limbs is utterly uncalled for when breed is valuable for stock purposes. With posterior extremity there may be a difference with horses, but in cattle I have amputated a hind limb with the best of satisfaction on two occasions. C. C. McLEAN, V. S.

MEADVILLE, PA.

DEATH FROM OVARIAN TUMOR.—I was recently called to the farm of Mr. Housington to visit a valuable mare, Winona ; eight years old ; record, 2:21. Found her very much distented in the abdomen, and a small anasarcous swelling along the belly ; the muscles of the thighs swollen considerably, but not presenting any of the characteristics of that along the lower portion of the abdomen. Temperature and pulse not much altered and no appearance of distress. Mr. H. informed me that two or three days previous to my visit she first began to enlarge rapidly.

This mare, with several others, had been in the hands of a driver on Long Island until a few months ago, and it was supposed by Mr. H that she had been turned to a horse, and was with foal, as some six or seven weeks previous to her death she began to enlarge somewhat, showing evidence of being seven or eight months with colt. About this time I was at the farm and my attention being called to the mare, I gave the opinion held by Mr. H. On seeing her the second time I still retained the opinion formed a month before, and without making an examination—as I was in very much of a hurry, having a case on my hands that required my immediate atttention—gave the opinion that she was suffering from dropsy of the uterus. When I visited her the following day, found her condition very much worse, terribly distended, large infiltration of serum into the abdominal and pectoral muscles, and those in the region of the thighs extending well down into the legs ; breathing not much affected ; pulse accelerated and weak ; temperature not taken.

Determined on making an examination of the uterus, dilating the os-uteri and evacuating the fluid, should it contain any ; but an examination revealed the uterus in an empty condition. Further examination per rectum, disclosed the presence of a massive tumor floating in a large accumulation of water in the abdominal cavity, which I diagnosed as an ovarian tumor.

During the course of the examination the mare went down, and shortly after, the skin and fascæ ruptured in the median line just anterior to the mammæ. She was now destroyed, and a post-mortem revealed the presence of a large ovarian tumor, twenty-nine and one half pounds in weight, while the abdominal cavity contained eight to ten pails of water.

The left ovary showed slight evidence of disease.

The mare, previous to coming into Mr. H—— ' hands, showed evidence of poor feed and care.

This case, I think, is interesting, considering the rarity of the disease in the mare, and particularly in one so young.

This animal, several months ago, would have offered a good opportunity for operation, as the attachment was very small, no adhesions, and could have been removed through a small opening, by evacuating with an aspirator, the contained fluid.

ADAMS, N. Y.

E. H. BRADLEY, V. S.

A CASE OF PARALYSIS OF THE BOWELS TREATED SUCCESSFULLY BY ELECTRICITY—The subject of this notice was an imported light bay entire horse. On the 9th of March I visited the horse which I found in a large, clean, well-aired box stall, lying stretched out, extremities cold, pulse 40, temp. 101 F., inspiration 12. On inquiry, I found the horse had not been fed regularly, by the same person, and that he was being fitted for the spring fairs, and consequently over-fed or suffuted.

TREATMENT.—1 oz. aloes barb., 2 dr. pot. carb., 2 dr. fl. ex. nux vomica, 2 dr. ginger. Injections of warm water and exercised. On the 11th there was no change for the better. I gave one qt. raw oil in addition to the nux vomica and the pot. carb., with hot blankets to abdomen and warm water and belladonna injections. 12th. No effect from the medicine. Had the abdomen well rubbed for an hour and brisk exercise for half an hour. Examination per rectum revealed nothing in reach. Continued the same treatment 13th and 14th. Horse in same condition; pulse, 60, inspiration, 20, temperature. 100, extremities warm. Gave one qt. beef tea, 20 gr quinine sulph., 1 dr. nux vomica, every six hours, and 1 pt. raw oil night and morning, and hot formations. This continued the 15th and 16th. Examinations per rectum, nothing reached. On 17th, concluded to give no more medicine, but to use electricity which was applied every six hours for twenty minutes at a time. On the afternoon of the 18th, just after using the battery, we were rewarded with a very copious fluid discharge which continued for twenty-four hours. Since then the horse has done well and after three weeks took first prize wherever exhibited.

NEW HAMBURG, N. Y. WM. STIRLING, V. S.

FRACTURE OF RADIUS IN A BULL.—I received a call to attend a Holstein bull, eighteen months old, weighing 1600 pounds, lying in the field with a transverse fracture in the middle of the radius. I applied a rope around the superior portion of the fractured bone and around the fetlock joint, with instructions to my assistants to pull steady until I adjusted the fractured end. I then bandaged, first with a dry bandage, and after the second wrapping covered with flour paste. I then placed two lateral splints, made of hard sole leather, to which was rivited two pieces of carriage-wheel tire, one inch wide, reaching above and below the fractured ends of the bone. A large external leather splint was placed over all. My reason for using paste instead of plaster of Paris is because the paste bandage is more readily removed after subsidence of inflammation. The bull, after lying four hours, got up and walked to a box stall. The day following he could walk and use the injured limb. One other case of fracture of radius and two of fracture of tibia, all in cattle, have made complete recovery with the same treatment.

HUNTINGTON, L. I. JOHN LINDSAY, D. V. S.

